# Functional Differentiation of *BnVTE4* Gene Homologous Copies in α-Tocopherol Biosynthesis Revealed by CRISPR/Cas9 Editing

**DOI:** 10.3389/fpls.2022.850924

**Published:** 2022-04-11

**Authors:** Haiyan Zhang, Yuqin Shi, Mengdan Sun, Xuezhi Hu, Mengyu Hao, Yu Shu, Xue-Rong Zhou, Qiong Hu, Chao Li, Desheng Mei

**Affiliations:** ^1^Oil Crops Research Institute of Chinese Academy of Agricultural Sciences, Key Laboratory for Biological Sciences and Genetic Improvement of Oil Crops, Ministry of Agriculture and Rural Affairs, Wuhan, China; ^2^Commonwealth Scientific and Industrial Research Organisation (CSIRO) Agriculture and Food, Canberra, ACT, Australia

**Keywords:** α-tocopherol, *BnVTE4*, homologous copies, functional diversification, CRISPR/Cas9

## Abstract

Tocopherols are essential nutrients for human health known as vitamin E. Vitamin E deficiency can have a profound effect on human health, including the central nervous system and cardiovascular and immune protection. Multiple enzymatic steps are involved in the conversion between different forms of tocopherols. Among them, γ-tocopherol methyltransferase encoded by gene *VTE4* catalyzes the conversion of γ- to α-tocopherol or δ- to β-tocopherol isoforms. However, the gene copies and their functional contribution of *VTE4* homologs in *Brassica napus* were not elucidated. To this end, different mutation combinations of four putative *BnVTE4* homologous copies were generated by using CRISPR/Cas9 genome editing technology. Editing of those *BnVTE4* homologs led to a significant change of the α-tocopherol content and the ratio between α- and γ-tocopherol compared with wide-type control. Analysis of the different combinations of *BnVTE4*-edited homologs revealed that the contribution of the *BnVTE4* individual gene displayed obvious functional differentiation in α-tocopherol biosynthesis. Their contribution could be in order of *VTE4.C02-2* (BnaC02G0331100ZS) > *VTE4.A02-1* (BnaA02G0247300ZS) > *VTE4.A02-2* (BnaA02G0154300ZS). Moreover, the *VTE4.A02-1* and *VTE4.A02-2* copies might have severe functional redundancies in α-tocopherol biosynthesis. Overall, this study systemically studied the different effects of *BnVTE4* homologs, which provided a theoretical basis for breeding high α-tocopherol content oilseed rape.

## Introduction

Tocopherols and tocotrienols are also called vitamin E. In this study, we focus on the analysis of tocopherols only. Tocopherols are lipid-soluble strong antioxidants that are involved in the protection of oxidative damage of membrane lipids by scavenging singlet oxygen and other reactive oxygen species (ROS) (Trebst et al., 2002; Schneider, 2005; Fritsche et al., 2017). Thus, tocopherols are presumed to be key scavengers of senescence or stress-induced lipid radicals and ROS in plants. Their strong antioxidant capacity can reduce lipid peroxy radicals to corresponding hydrogen peroxide to avoid lipid peroxidation of polyunsaturated fatty acids. The high antioxidative property exerts a protective role in multiple plant stress responses such as cold and drought stress (Janeczko et al., 2018; Ma et al., 2020). Most importantly, this effect can also be extended to human health. Vitamin E is an essential nutrient in the human daily diet. Vitamin E deficiency primarily causes muscle atrophy and reproductive and neurologic dysfunctions (Martin et al., 2013; Kumar et al., 2018), whereas the adequate intake of vitamin E can prevent neurological diseases, cataracts, coronary heart disease, atherosclerosis, diabetes, Parkinson’s disease, Alzheimer’s disease, and vision diseases (Lloret et al., 2019; Rozanowska et al., 2019). Thus, biofortification of vitamin E in crop plants is not only beneficial for human health but also for plant stress response.

Based on the different numbers and positions of methyl substituents on the aromatic ring, tocopherols are defined as four isoforms, namely α-, β-, γ-, and δ-tocopherol (α-T, β-T, γ-T, and δ-T in short). Although those tocopherols have similar antioxidant activities *in vitro*, the vitamin E activity *in vivo* is significantly different. Among them, α-T possesses the highest vitamin E activity *in vivo* and is also a common type of tocopherol in the European diet (Galmés et al., 2018). It has been proven that α-T supplementation can improve cell-mediated immunity, and only α-T is selected to set the recommended dietary allowance (RDA) of vitamin E for Americans (Meydani et al., 1990; Meydani et al., 1997; Institute of Medicine (US) Panel on Dietary Antioxidants and Related Compounds, 2000; Ranard and Erdman, 2018). Tocopherols are only synthesized on the inner chloroplast membrane of photosynthetic organisms, including plants, green algae, and some cyanobacteria. Undoubtedly, a daily supplement of α-T derived from plant-based food is a safe and natural way to ensure human health.

Oilseed rape (*Brassica napus* L., AACC, 2n = 38) is one of the most important resources of edible vegetable oil in the world, accounting for about 16% of the total global vegetable oil production (Cheng et al., 2019). High-quality rapeseed oil, containing multiple beneficial nutrients including vitamin E, is the dominant edible vegetable oil in China, Europe, and Canada. A daily supplement of vitamin E *via* rapeseed oil is the safest and most effective way to keep the nutritional requirement for the human body. Therefore, genetic improvement of vitamin E content has been considered a key breeding objective (Cheng et al., 2019; Yan et al., 2021). However, oilseed rape is a typical allotetraploid crop, and most of the genes have multiple homologous copies with redundant or diverse functions (Chalhoub et al., 2014; Braatz et al., 2017; Zaman et al., 2019, 2021; Li et al., 2021). Clarification of the detailed roles of each homologous copy is the basis of the further breeding application.

**FIGURE 1:**
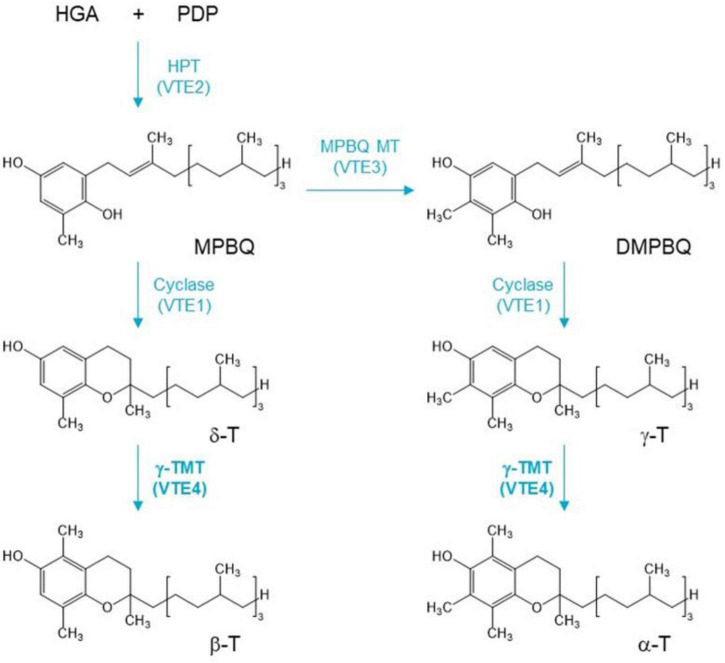
A simplified pathway of vitamin E isoform conversion. HGA, homogentisic acid; PDP, phytyldiphosphate; MPBQ, 2-methyl-6-phytyl-1,4-benzoquinol; DMPBQ, 2,3-dimethyl-6-phytyl-1,4-benzoquinone; α-T, β-T, γ-T, and δ-T are α, β, γ, and δ isoforms of tocopherol. *VTE* genes are described in the text. γ-Tocopherol methyltransferase (γ-TMT) gene *VTE4* (bold) is the target gene to be edited in this study.

The biosynthesis pathway of tocopherols has been extensively studied in the model plant *Arabidopsis thaliana* (Valentin et al., 2006; Vom Dorp et al., 2015). Tocopherol biosynthesis begins with the formation of homogentisic acid (HGA), which is catalyzed by p-hydroxyphenylpyruvic acid dioxygenase (HPPD/PDS1) and is derived from the deamination of tyrosine (Norris et al., 1995, 1998; Tsegaye et al., 2002). Phytyldiphosphate (PDP) is formed by phytol kinase, and phytyl—P kinase catalyzes the formation of phytol (Valentin et al., 2006; Vom Dorp et al., 2015). HGA and PDP are condensed to 2-methyl-6-phytyl-1,4-benzoquinol (MPBQ) catalyzed by the enzyme homogentisatephytyltransferase (HPT), which is encoded by *VTE2* gene (Venkatesh et al., 2006). The 2-methyl-6-phyty-1,4-benzoquinol methyltransferase (MPBQ MT) methylates MPBQ to 2,3-dimethyl-6-phytyl-1,4-benzoquinone (DMPBQ), while MPBQ and DMPBQ are transformed into γ- and δ-tocopherol, respectively, by tocopherol cyclase (TC). TC is encoded by the *VTE1* gene, and MPBQ MT is encoded by *VTE3* gene (Porfirova et al., 2002; Semchuk et al., 2009). Then, the γ-Tmethyltransferase (γ-TMT) encoded by *VTE4* gene catalyzes the conversion of δ- to β-T and γ- to α-T (Porfirova et al., 2002; Bergmüller et al., 2003; DellaPenna and Pogson, 2006; Hunter and Cahoon, 2007; Figure 1). Thus, *VTE4* directly affects the content of α-T and γ-T. However, the putative functional differentiation of the *VTE4* gene in *B. napus* is still unclear.

In this study, four putative copies of the *BnVTE4* gene have been identified from the latest oilseed rape genome database. The functional contribution of *BnVTE4* homologs in the α-T biosynthesis is studied by generating different mutation types using CRISPR/Cas9 genome editing technology. This study will shed new light on the breeding application of high α-T content in oilseed rape.

## Materials and Methods

### sgRNA Design and Vector Construction

Four homologous copies of the *BnVTE4* gene were retrieved in the *B. napus* genome database,^1^ namely BnaC02G0197500ZS (VTE4.C02-1), BnaC02G0331100ZS (VTE4.C02-2), BnaA02G0247300ZS (VTE4.A02-1), and BnaA02G015430ZS (VTE4.A02-2). Two sgRNAs with minimal off-target effects were designed using CRISPR-P 2.0^2^ at the conserved sequence positions of the third and fourth exons, namely S1 (GGTGAGCATATGCCTGACA) and S2 (CCATGGGAGCAGAACCTCT). The sgRNA assembly and vector construction were performed as a previous report (Xing et al., 2014; Ma et al., 2015).

### Plant Material and Genetic Transformation of Oilseed Rape

The qualified genome editing vector was transferred into *Agrobacterium tumefaciens* strain (GV3101) by the heat shock method. The vector containing two sgRNAs was introduced into *B. napus* L. variety “Zhongshuang 6” by the *Agrobacterium*-mediated transformation (Li et al., 2018). The selection marker was Kanamycin. The T_0_ generation mutants were planted in the artificial climate room and grown under a photoperiod of the 16 h light/8 h dark at a temperature of 22°C, and the T_1_ generation was planted in the field of the Hanchuan transgenic base, Hubei, China.

### Identification of Positive Mutants

Plant genomic DNA was extracted from leaves by the CTAB (hexadecyltriethyl ammonium bromide) method. We used *NPTII* gene-specific primers NPTII-F (5′-GATGGATTGCACGCAGGT-3′) and NPTII-R (5′-TCGTCAAGAAGGCGATAGA-3′) for PCR reaction to identify positive transgenic plants.

To identify whether the *BnVTE4* gene of the positive transgenic plants had been edited, gene-specific primers (Supplementary Table 1) were used to amplify the DNA sequence containing the target site by PCR, and then Sanger sequencing was used to identify the mutants. The heterozygous mutants were determined by the Hi-TOM platform (Liu et al., 2019). Hi-TOM sequencing consists of two rounds of PCR. In the first round of PCR, gene-specific primers (Supplementary Table 1) were used to amplify the genomic sequence of about 500–2,000 bp around the 4 copies of the target site. In the second round of PCR, gene-specific primers containing Hi-TOM adaptor primers (Supplementary Table 1) were used to amplify the 80–300 bp genomic region around the target site. The products of the second round were sequenced by the company.^3^

### Tocopherol Extraction and Analysis

Tocopherol extraction was performed according to the reported method with slight modification (Yu et al., 2016; Xu et al., 2019). A total of 200 mg seeds were placed in a 2 ml centrifuge tube with 1 steel bead of a 5-mm diameter and grounded for 5 min at 60 Hz using a rapid grinder. An accurate 60 mg aliquot was weighed from the ground seeds. Three biological replicates were set up. Tocopherols were extracted by adding 1.5-ml hexane. The mixture was sealed and shaken for 4 h in the dark and then extracted at 4°C for 12 h. The mixture was centrifuged at 10,000 rpm for 10 min, and the supernatant was filtered through a.22-μm organic membrane.

Determination of tocopherols was carried out on high-performance liquid chromatography (HPLC, Waters). Agilent liquid chromatography column ZORBAX RX-SIL (4.6 mm × 250 mm) was used, and the temperature was set at 30°C. The mobile phase was n-Hexane:isopropanol (99:1, v/v) at a flow rate of 1 ml/min. The sample composition was determined qualitatively and quantitatively by UV light at 292 nm. Standards (95%, pure HPLC) for α- and γ-T were purchased from Merck, and all the standards and samples were in 5 μl injection volumes.

Statistical software SPSS v22.0 was used to analyze the data, and one-way ANOVA was employed to comparatively analyze the differences between α- and γ-T of different copy mutation combination materials and wild-type rape seeds (*p* < 0.05).

## Results

### Sequence Analysis and Vector Construction for *BnVTE4* Gene

**FIGURE 2:**
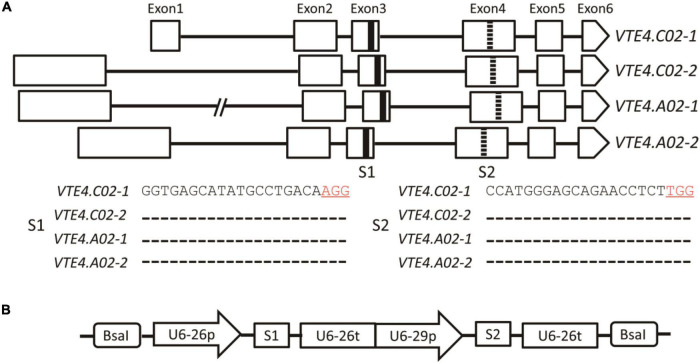
A schematic diagram of *BnVTE4* gene editing vector construction. **(A)** Rectangular boxes indicate exons of *VTE4.C02-1*, *VTE4.C02-2*, *VTE4.A02-1*, and *VTE4.A02-2* copies; black horizontal lines represent introns; thickened black vertical lines show sgRNA1 (S1) targeting Exon3; thickened dashed lines indicate sgRNA2 (S2) targeting Exon4. **(B)** A schematic diagram of the *BnVTE4* gene editing vector. sgRNA1 is initiated and terminated by U6-26p and U6-26t, respectively, sgRNA2 is initiated and terminated by U6-29p and U6-26t, respectively.

Genomic sequence analysis showed that the *BnVTE4* gene possesses four homologous copies in *B. napus*, each of which is composed of six exons and five introns. Two sgRNAs (named S1 and S2) were designed in their conserved sequence regions located in the third and fourth exons, respectively (Figures 2A,B).

### Generation of Different *BnVTE4* Mutation Types in Oilseed Rape

**FIGURE 3:**
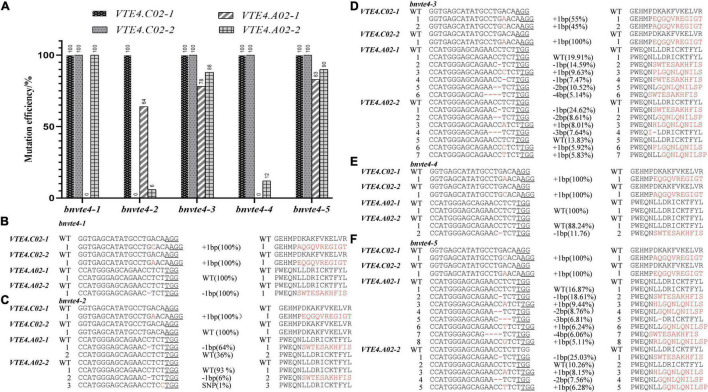
Mutation type, mutation efficiency, and amino acid alterations in homologous copies of the *BnVTE4* gene in the T_1_ generation. **(A)** Mutation efficiency of each copy of the *BnVTE4* mutant. **(B)** Mutation types and amino acid alterations in homologous copies in the *BnVTE4* gene of *bnvte4-1*. **(C)** Mutation types and amino acid alterations in homologous copies in the *BnVTE4* gene of *bnvte4-2.* **(D)** Mutation types and amino acid alterations in homologous copies in the *BnVTE4* gene of *bnvte4-3.* **(E)** Mutation types and amino acid alterations in homologous copies in the *BnVTE4* gene of *bnvte4-4*. **(F)** Mutation types and amino acid alterations in homologous copies in the *BnVTE4* gene of *bnvte4-5.*

In order to elucidate the possible functional differentiation of different homologous copies of the *BnVTE4* gene during α-T synthesis, we screened different mutation types of *BnVTE4* editing in the T_1_ generation. Five editing types with different mutation combinations were obtained, named *bnvte4-1, bnvte4-2*, *bnvte4-3*, *bnvte4-4*, and *bnvte4-5*, respectively. Sequencing results indicated that *VTE4.C02-1* and *VTE4.C02-2* copies were homozygous mutations or wild type (WT) (Figure 3A). All five editing types had a homozygous mutation in the *VTE4.C02-1* copy with a single base insertion leading to a frame shift. The *VTE4.C02-2* copy also had homozygous mutations with single-base insertions leading to a frame shift in *bnvte4-1, bnvte4-3*, *bnvte4-4*, and *bnvte4-5*, except for *bnvte4-2* that was not mutated (Figure 3). *VTE4.A02-1* and *VTE4.A02-2* copies had heterozygous mutations in some editing types, and Hi-TOM high-throughput sequencing was employed to verify the editing frequency and amino acid changes at the targeted sites (Figures 3B–F and Supplementary Figures 1, 2). *VTE4.A02-1* in *bnvte4-1* and *bnvte4-4* was unmutated (WT). One nucleotide deletion in the *VTE4.A02-1* copy of *bnvte4-2* caused 64% of the frame shifts (Figures 3A,C). Both *bnvte4-3* and *bnvte4-5* had one or several nucleotide deletions and one nucleotide insertion in the *VTE4.A02-1* copy, 78 and 83%, respectively (Figures 3A,D,F), resulting in frame shifts or amino acid deletions. The *VTE4.A02-2* copies of *bnvte4-1* had a deletion of one nucleotide, resulting in frame shifts (Figures 3A,B). The *VTE4.A02-2* copies of *bnvte4-2* had a deletion of one nucleotide, resulting in frame shifts that accounted for only 6%, and the one nucleotide substitution without amino acid change accounted for only 1% (Figures 3A,C). Both *bnvte4-3* and *bnvte4-5* had one or more nucleotide deletion and one nucleotide insertion in the *VTE4.A02-2* copy, accounting for 88 and 90%, respectively (Figures 3A,D,F), resulting in frame shifts or amino acid deletions. The *VTE4.A02-2* copy of *bnvte4-4* had one or more nucleotide deletions, resulting in frame shifts of just 12% (Figures 3A,E).

### Determination of Tocopherol Content in *BnVTE4* Mutant Types

**FIGURE 4:**
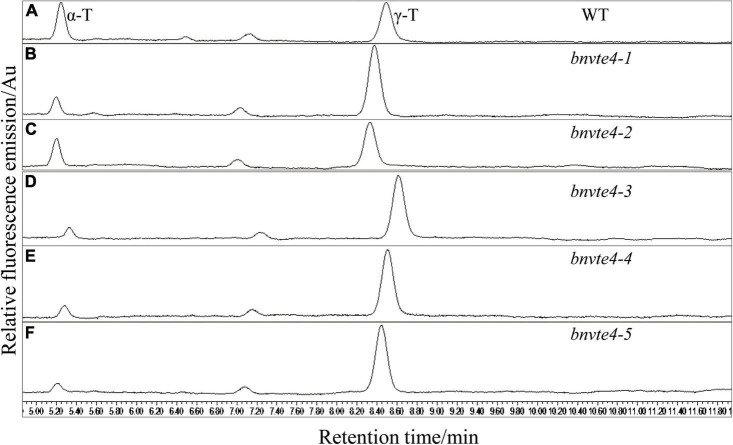
Determination of the components and content of tocopherol by HPLC. The peak at 5.2 min indicated α-T, the peak at 8.5 min indicated γ-T and the peak area indicated the content of tocopherol. **(A)** Two types of tocopherols in the wild type control (WT) were determined by HPLC. **(B)** Two types of tocopherols in *bnvte4-1* were determined. **(C)** Two types of tocopherols in *bnvte4-2* were determined. **(D)** Two types of tocopherols in *bnvte4-3* were determined. **(E)** Two types of tocopherols in *bnvte4-4* were determined. **(F)** Two types of tocopherols in *bnvte4-5* were determined.

The five *BnVTE4* T_1_-mutated lines and WT control were grown and harvested under the same condition with good growth and no significant difference from the control (Supplementary Figure 3), and mature seeds were used to determine the content and composition of tocopherols. HPLC results showed that the contents of α-T and γ-T were successfully detected in both WT control and T_1_ seeds (Figure 4). Consistent with a previous report (Zhang et al., 2007), the β- and δ-tocopherol contents were extremely low, which were neglected in the subsequence analysis.

**FIGURE 5:**
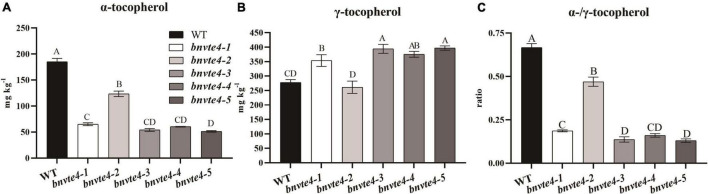
The α-and γ-T content and the ratio in T_1_ transgenic mutants. **(A)** The α-T content of the mutants. **(B)** The γ-T content of the mutants. **(C)** The α-/γ-T ratios of mutants.

As shown in Figure 4, α-T content in *BnVTE4* mutant lines was substantially decreased and significantly lower than that in WT (*p* < 0.05, Figure 5A). The reduction of α-T content in the *bnvte4-2* mutant type was the lowest one among these five mutant types. The reduced α-T content was accompanied by a significant increase in γ-T content compared to the WT (*p* < 0.05, Figure 5B), except for the *bnvte4-2* mutant type, which had a similar level of γ-T compared to WT. Nevertheless, the ratios of α- to γ-T (α-/γ-T) in all five *BnVTE4* mutant types were only 0.1– 0.5, which were significantly lower than 0.67 in WT oilseed rape (*p* < 0.05, Figure 5C). This result confirmed that the mutations in the *BnVTE4* gene can significantly affect the conversion of γ-T to α-T.

Comparing the mutations in individual copies of the *BnVTE4* gene (Figure 3A) with the change in tocopherol composition (Figure 5), we found that the homologous copies of the *BnVTE4* gene had different contributions in α-T biosynthesis. *bnvte4-3* and *bnvte4-5* lines had both homozygous mutations in *VTE4.C02-1* and *VTE4.C02-2* copies, and the editing efficiency in *VTE4.A02-1* and *VTE4.A02-2* copies were both more than 50%. There was no significant difference in α-T and γ-T content, as well as the α-/γ-T ratio between these two lines. The *bnvte4-1* and *bnvte4-5* differed in the mutation of the *VTE4.A02-1* copy. The *bnvte4-1* had no mutation in *VTE4.A02-1*, while *bnvte4-5* had 83% editing efficiency in this gene. The *bnvte4-1* showed significant differences in α- and γ-T contents and the α-/γ-T ratio compared with *bnvte4-5*, implying the contribution of the *VTE4.A02-1* copy to α-T synthesis.

The *bnvte4-1* and *bnvte4-4* were only different at the mutation in the *VTE4.A02-2* copy. The *VTE4.A02-2* copy in *bnvte4-1* was completely mutated, while the editing efficiency of *bnvte4-4* was only 12%, but the α-T and γ-T contents and the α-/γ-T ratio were not significantly different. This suggested that the contribution of the *VTE4.A02-2* copy might be low. Alternatively, the wild-type copy of *VTE4* in *bnvte4-1* and *bnvte4-4* might dominate the contribution, leading to the effect of the *VTE4.A02-2* mutant being insignificant.

*VTE4.C02-1* and *VTE4.A02-2* copies in *bnvte4-2* and *bnvte4-4* had similar mutation patterns, while the other two *VTE4* genes had different mutation profiles. The *VTE4.C02-2* copy in *bnvte4-2* was WT, and its *VTE4.A02-1* copy had an editing efficiency of 64%, while *VTE4.C02-2* in *bnvte4-4* was completely mutated, and its *VTE4.A02-1* copy was not mutated. The α-T and γ-T contents and the α-/γ-T ratio between *bnvte4-2* and *bnvte4-4* were significantly different. The α-/γ-T ratio of *bnvte4-2* was higher than that of *bnvte4-4*, suggesting that the contribution of *VTE4.C02-2* might be greater than that of *VTE4.A02-1*.

The α-T and γ-T contents, and the α-/γ-T ratio of *bnvte4-3*, *bnvte4-4*, and *bnvte4-5* were not significantly different. In terms of mutation type, *bnvte4-3* and *bnvte4-5* were similar. *VTE4.C02-1* and *VTE4.C02-2* copies in *bnvte4-4* were fully mutated, same as in *bnvte4-3* and *bnvte4-5*. However, *VTE4.A02-1* and *VTE4.A02-2* copies did not exhibit mutation type, which again demonstrated that *VTE4.A02-1* and *VTE4.A02-2* copies might have a minor contribution.

It is particularly worth mentioning the obviously different tocopherol profile of *bnvte4-2* to other mutant types. *bnvte4-2* had similar mutations to other lines except for the *VTE4.C02-2* copy that was WT other than homozygous mutation in other lines. Considering the lowest reduction of α-T in *bnvte4-2* (from 185 mg/kg in WT to 123 mg/kg), while other lines had reduced α-T to ∼60 mg/kg, the key difference was the WT copy of *VTE4.C02-2* compared to a homozygous mutation in other lines. This again suggested the great contribution of *VTE4.C02-2* in α-T synthesis.

## Discussion

Tocopherol, especially α-tocopherol, is not only an important scavenger of stress-induced oxidative damage but an essential nutrient for human health. Genetic improvement of high-quality rapeseed oil with multiple vitamins such as α-tocopherol is an effective way to ensure the daily nutritional requirement of the human body. However, the complex genomic structure of oilseed rape that most of the genes have multiple homologous gene copies with putative redundant or diverse gene functions is one of the open questions for breeding application.

CRISPR/Cas9 technology has several inherent advantages in the precisely studying gene functions and subsequent application in crop plants (Lee et al., 2018; Hirohata et al., 2019; Zhai et al., 2019), especially polyploidy crops such as oilseed rape. In our previous studies, the highly efficient genome editing platform has been established to dissect the functional diversity of different homologs in oilseed rape (Li et al., 2018; Zaman et al., 2019; Cheng et al., 2021). Many independent case studies suggested that the homologous gene copies, although possess similar genomic information, often exert different effects in a particular trait (Okuzaki et al., 2018; Zhai et al., 2019, 2020; Ahmar et al., 2021; Chen et al., 2021).

To elucidate the contribution of the *BnVTE4* gene in α-T biosynthesis, its four homologous gene copies were studied in detail by using CRISPR/Cas9 genome technology. To generate different mutation combinations of *BnVTE4* homologs, two sgRNAs were designed to target their conserved regions in exon 3 and exon 4 of the coding sequences. Sequencing data demonstrated that the majority of *BnVTE4*-mutated lines showed homozygous mutation types in *VTE4.C02-1* and *VTE4.C02-2* homologous copies (Figure 3A), which suggested that our designed sgRNA1 had high mutation capacity on the genomic region of *BnVTE4* homologs. However, it was very difficult to obtain homozygous mutation types in *VTE4.A02-1* and *VTE4.A02-2* homologs, even dozens of positive transgenic T_0_ lines and plenty of T_2_ plants had been performed by mutation screening. One of our hypotheses is that those four homologous copies probably have functional diversification in α-tocopherol biosynthesis or other key developmental processes such as seed vigor. Similar to this result, simultaneous mutation of five *BnJAG* homologs drastically affected the seed development, and its seeds are hard to survive, whereas the single mutation of *BnJAG.A08-NUB* homologous copy displays a pod-shattering resistance phenotype (Zaman et al., 2019).

To further evaluate the contribution of *BnVTE4* homologs in α-tocopherol biosynthesis, the mutation frequency of *VTE4.A02-1* and *VTE4.A02-2* homologs was quantified by the Hi-TOM high-throughput sequencing method. Hi-TOM data suggested that the mutation frequency of *VTE4.A02-1* and *VTE4.A02-2* homologous copies exhibited a significant difference in *bnvte4-1* and *bnvte4-5* mutation lines. In addition, sequencing data showed that the most common mutation types were –1/+1 bp indels, and the amino acid sequence was completely changed due to a frame shift (Figures 3B–F). Thus, the designed sgRNAs can effectively generate targeted mutagenesis in all *BnVTE4* homologous copies. This result was further verified by subsequence analysis of tocopherols content using HPLC, which demonstrated that the α- and γ-tocopherol contents and α-/γ-tocopherol ratios of these mutated lines showed significant alteration compared to WT control.

However, the effect of different mutation combinations on α-T content was a significant difference among five mutated lines. Similar mutation types (*bnvte4-3* and *bnvte4-5*) showed no significant difference in α-T content. There was no significant difference in the content of α-T when the mutation types of the other copies except *VTE4.A02-2* were similar (*bnvte4-1* and *bnvte4-4*), indicating that the *VTE4.A02-2* copy did not play a major role in α-tocopherol biosynthesis. This conclusion was supported by comparing *bnvte4-3* and *bnvte4-5*. The content of α-T was significantly different when the mutation types were similar for other copies except for *VTE4.A02-1* (*bnvte4-1* and *bnvte4-5*), indicating the *VTE4.A02-1* copy was important. *VTE4.C02-2* and *VTE4.A02-2* mutation types of *bnvte4-2* and *bnvte4-3* were different, and the content of α-T was significantly varied. The functionality of the *VTE4.C02-2* copy was revealed in *BnVTE4-2* by comparing it to other lines. The significant difference in the content of α-T when the mutation types of other copies except *VTE4.C02-2* are similar (*bnvte4-2* and *bnvte4-4*) indicates that *VTE4.C02-2* played an important role. Taken together, those data demonstrated that the four *BnVTE4* gene homologs might have functional differentiation in α-T biosynthesis, and their contribution was likely VTE4.C02-2 (BnaC02G0331100ZS) > VTE4.A02-1 (BnaA02G0247300ZS) > VTE4.A02-2 (BnaA02G0154300ZS). This knowledge will shed new light on the cultivation of high α-T-content oilseed rape.

## Data Availability Statement

The datasets presented in this study can be found in online repositories. The names of the repository/repositories and accession number(s) can be found in the article/Supplementary Material.

## Author Contributions

QH, X-RZ, CL, and DM conceived and revised the manuscript. CL designed the experiments. HZ, YQS, MS, XH, MH, and YS performed the experiments. HZ and CL analyzed the data. HZ drafted the manuscript. All authors reviewed and approved the manuscript.

## Conflict of Interest

The authors declare that the research was conducted in the absence of any commercial or financial relationships that could be construed as a potential conflict of interest.

## Publisher’s Note

All claims expressed in this article are solely those of the authors and do not necessarily represent those of their affiliated organizations, or those of the publisher, the editors and the reviewers. Any product that may be evaluated in this article, or claim that may be made by its manufacturer, is not guaranteed or endorsed by the publisher.

## Footnotes

1
http://cbi.hzau.edu.cn/bnapus/

2
http://crispr.hzau.edu.cn/cgi-bin/CRISPR2/CRISPR

3
http://121.40.237.174/Hi-TOM/

## References

[B1] Ahmar S. Zhai Y. Huang H. Yu K. Khan M. H. U. Shahid M. (2021). Development of mutants with varying flowering times by targeted editing of multiple SVP gene copies in *Brassica napus* L. *Crop J.* 10 67–74. 10.1016/j.cj.2021.03.023

[B2] Bergmüller E. Porfirova S. DöRmann P. (2003). Characterization of an *Arabidopsis* mutant deficient in γ-tocopherol methyltransferase. *Plant Mol. Biol.* 52 1181–1190. 10.1023/b:plan.0000004307.62398.91 14682617

[B3] Braatz J. Harloff H. J. Mascher M. Stein N. Himmelbach A. Jung C. (2017). CRISPR-Cas9 targeted mutagenesis leads to simultaneous modification of different homoeologous gene copies in polyploid oilseed rape (*Brassica napus*). *Plant Physiol.* 174 935–942. 10.1104/pp.17.00426 28584067 PMC5462057

[B4] Chalhoub B. Denoeud F. Liu S. Parkin I. A. Tang H. Wang X. (2014). Plant genetics. Early allopolyploid evolution in the post-Neolithic Brassica napus oilseed genome. *Science* 345 950–953. 10.1126/science.1253435 25146293

[B5] Chen Y. Fu M. Li H. Wang L. Liu R. Liu Z. (2021). High-oleic acid content, nontransgenic allotetraploid cotton (*Gossypium hirsutum* L.) generated by knockout of GhFAD2 genes with CRISPR/Cas9 system. *Plant Biotechnol. J.* 19 424–426. 10.1111/pbi.13507 33131175 PMC7955888

[B6] Cheng H. Hao M. Ding B. Mei D. Wang W. Wang H. (2021). Base editing with high efficiency in allotetraploid oilseed rape by A3A-PBE system. *Plant Biotechnol. J.* 19 87–97. 10.1111/pbi.13444 32640102 PMC7769242

[B7] Cheng L. Zhong-Chao F. Tang-Hua X. Xiao-Min M. Guang-Sheng Z. FengHong H. (2019). Development, potential and adaptation of Chinese rapeseed industry. *Chin. J. of Oil Crop Sci.* 41 485–489.

[B8] DellaPenna D. Pogson B. J. (2006). Vitamin synthesis in plants: tocopherols and carotenoids. *Annu. Rev. Plant Biol.* 57 711–738. 10.1146/annurev.arplant.56.032604.144301 16669779

[B9] Fritsche S. Wang X. Jung C. (2017). Recent advances in our understanding of tocopherol biosynthesis in plants: an overview of key genes, functions, and breeding of vitamin E improved crops. *Antioxidants* 6:99. 10.3390/antiox6040099 29194404 PMC5745509

[B10] Galmés S. Serra F. Palou A. (2018). Vitamin E metabolic effects and genetic variants: a challenge for precision nutrition in obesity and associated disturbances. *Nutrients* 10:1919. 10.3390/nu10121919 30518135 PMC6316334

[B11] Hirohata A. Sato I. Kaino K. Iwata Y. Koizumi N. Mishiba K. I. (2019). CRISPR/Cas9-mediated homologous recombination in tobacco. *Plant Cell Rep.* 38 463–473. 10.1007/s00299-018-2320-7 30006757

[B12] Hunter S. C. Cahoon E. B. (2007). Enhancing vitamin E in oilseeds: unraveling tocopherol and tocotrienol biosynthesis. *Lipids* 42 97–108. 10.1007/s11745-007-3028-6 17393215

[B13] Institute of Medicine (US) Panel on Dietary Antioxidants and Related Compounds (2000). *Dietary Reference Intakes for Vitamin C, Vitamin E, Selenium, and Carotenoids.* Washington, DC: National Academies Press (US). 25077263

[B14] Janeczko A. Dziurka M. Pociecha E. (2018). Increased leaf tocopherol and β-carotene content is associated with the tolerance of winter wheat cultivars to frost. *J. Agron. Crop Sci.* 204 594–602. 10.1111/jac.12287

[B15] Kumar D. A. Kumar J. S. Vignesh M. Uttamrao Z. R. Singh C. H. Gulab C. (2018). Molecular diversity and genetic variability of kernel tocopherols among maize inbreds possessing favourable haplotypes of γ-tocopherol methyl transferase (ZmVTE4). *J. Plant Biochem. Biotechnol.* 28 253–262. 10.1007/s13562-018-0470-x

[B16] Lee Z. H. Yamaguchi N. Ito T. (2018). Using CRISPR/cas9 system to introduce targeted mutation in *Arabidopsis*. *Methods Mol Biol.* 1830 93–108. 10.1007/978-1-4939-8657-6_6 30043366

[B17] Li C. Hao M. Wang W. Wang H. Chen F. Chu W. (2018). An Efficient CRISPR/Cas9 platform for rapidly generating simultaneous mutagenesis of multiple gene homoeologs in allotetraploid oilseed rape. *Front. Plant Sci.* 9:442. 10.3389/fpls.2018.00442 29731757 PMC5920024

[B18] Li C. Sang S. Sun M. Yang J. Shi Y. Hu X. (2021). Direct modification of multiple gene homoeologs in *Brassica oleracea* and *Brassica napus* using doubled haploid inducer-mediated genome-editing system. *Plant Biotechnol. J.* 19 1889–1891. 10.1111/pbi.13632 34009735 PMC8486254

[B19] Liu Q. Wang C. Jiao X. Zhang H. Song L. Li Y. (2019). Hi-TOM: a platform for high-throughput tracking of mutations induced by CRISPR/Cas systems. *Sci. China Life Sci.* 62 1–7. 10.1007/s11427-018-9402-9 30446870

[B20] Lloret A. Esteve D. Monllor P. Cervera-Ferri A. Lloret A. (2019). The effectiveness of vitamin E treatment in Alzheimer’s disease. *Int J. Mol. Sci.* 20:879. 10.3390/ijms20040879 30781638 PMC6412423

[B21] Ma J. Qiu D. Gao H. Wen H. Wu Y. Pang Y. (2020). Over-expression of a γ-tocopherol methyltransferase gene in vitamin E pathway confers PEG-simulated drought tolerance in alfalfa. *BMC Plant Biol.* 20:226. 10.1186/s12870-020-02424-1 32429844 PMC7238615

[B22] Ma X. Zhang Q. Zhu Q. Liu W. Chen Y. Qiu R. (2015). A Robust CRISPR/Cas9 system for convenient, high-efficiency multiplex genome editing in monocot and dicot plants. *Mol. Plant* 8 1274–1284. 10.1016/j.molp.2015.04.007 25917172

[B23] Martin C. Zhang Y. Tonelli C. Petroni K. (2013). Plants, diet, and health. *Annu. Rev. Plant Biol.* 64 19–46. 10.1146/annurev-arplant-050312-120142 23451785

[B24] Meydani S. N. Barklund M. P. Liu S. Meydani M. Miller R. A. Cannon J. G. (1990). Vitamin E supplementation enhances cell-mediated immunity in healthy elderly subjects. *Am. J. Clin. Nutr.* 52 557–563. 10.1093/ajcn/52.3.557 2203257

[B25] Meydani S. N. Meydani M. Blumberg J. B. Leka L. S. Siber G. Loszewski R. (1997). Vitamin E supplementation and *in vivo* immune response in healthy elderly subjects. A randomized controlled trial. *JAMA* 277 1380–1386. 10.1001/jama.1997.03540410058031 9134944

[B26] Norris S. R. Barrette T. R. Dellapenna D. (1995). Genetic dissection of carotenoid synthesis in arabidopsis defines plastoquinone as an essential component of phytoene desaturation. *Plant Cell* 7 2139–2149. 10.1105/tpc.7.12.2139 8718624 PMC161068

[B27] Norris S. R. Shen X. Dellapenna D. (1998). Complementation of the *Arabidopsis* pds1 mutation with the gene encoding p-hydroxyphenylpyruvate dioxygenase. *Plant Physiol* 117 1317–1323. 10.1104/pp.117.4.1317 9701587 PMC34895

[B28] Okuzaki A. Ogawa T. Koizuka C. Kaneko K. Inaba M. Imamura J. (2018). CRISPR/Cas9-mediated genome editing of the fatty acid desaturase 2 gene in Brassica napus. *Plant Physiol. Biochem.* 131 63–69. 10.1016/j.plaphy.2018.04.025 29753601

[B29] Porfirova S. Bergmuller E. Tropf S. Lemke R. Dormann P. (2002). Isolation of an *Arabidopsis* mutant lacking vitamin E and identification of a cyclase essential for all tocopherol biosynthesis. *Proc. Natl. Acad. Sci. U.S.A.* 99 12495–12500. 10.1073/pnas.182330899 12213958 PMC129473

[B30] Ranard K. M. Erdman J. W. Jr. (2018). Effects of dietary RRR α-tocopherol vs all-racemic α-tocopherol on health outcomes. *Nutr. Rev.* 76 141–153. 10.1093/nutrit/nux067 29301023

[B31] Rozanowska M. Edge R. Land E. J. Navaratnam S. Sarna T. Truscott T. G. (2019). Scavenging of retinoid cation radicals by urate, trolox, and α-, β-, γ-, and δ-tocopherols. *Int. J. Mol. Sci.* 20:2799. 10.3390/ijms20112799 31181693 PMC6600601

[B32] Schneider C. (2005). Chemistry and biology of vitamin E. *Mol. Nutr. Food Res.* 49 7–30. 10.1002/mnfr.200400049 15580660

[B33] Semchuk N. M. Lushchak O. V. Falk J. Krupinska K. Lushchak V. I. (2009). Inactivation of genes, encoding tocopherol biosynthetic pathway enzymes, results in oxidative stress in outdoor grown *Arabidopsis thaliana*. *Plant Physiol. Biochem.* 47 384–390. 10.1016/j.plaphy.2009.01.009 19264498

[B34] Trebst A. Depka B. Holländer-Czytko H. (2002). A specific role for tocopherol and of chemical singlet oxygen quenchers in the maintenance of photosystem II structure and function in *Chlamydomonas reinhardtii*. *FEBS Lett.* 516 156–160. 10.1016/s0014-5793(02)02526-7 11959123

[B35] Tsegaye Y. Shintani D. K. Dellapenna D. (2002). Overexpression of the enzyme p-hydroxyphenolpyruvate dioxygenase in *Arabidopsis* and its relation to tocopherol biosynthesis. *Plant Physiol. Biochem.* 40 913–920. 10.1016/s0981-9428(02)01461-4

[B36] Valentin H. E. Lincoln K. Moshiri F. Jensen P. K. Qi Q. Venkatesh T. V. (2006). The *Arabidopsis* vitamin E pathway gene5-1 mutant reveals a critical role for phytol kinase in seed tocopherol biosynthesis. *Plant Cell* 18 212–224. 10.1105/tpc.105.037077 16361393 PMC1323494

[B37] Venkatesh T. V. Karunanandaa B. Free D. L. Rottnek J. M. Baszis S. R. Valentin H. E. (2006). Identification and characterization of an *Arabidopsis* homogentisate phytyltransferase paralog. *Planta* 223 1134–1144. 10.1007/s00425-005-0180-1 16408209

[B38] Vom Dorp K. Hölzl G. Plohmann C. Eisenhut M. Abraham M. Weber A. P. (2015). Remobilization of phytol from chlorophyll degradation is essential for tocopherol synthesis and growth of *Arabidopsis*. *Plant Cell* 27 2846–2859. 10.1105/tpc.15.00395 26452599 PMC4682318

[B39] Xing H. L. Dong L. Wang Z. P. Zhang H. Y. Han C. Y. Liu B. (2014). A CRISPR/Cas9 toolkit for multiplex genome editing in plants. *BMC Plant Biol.* 14:327. 10.1186/s12870-014-0327-y 25432517 PMC4262988

[B40] Xu J. Nwafor C. C. Shah N. Zhou Y. Zhang C. (2019). Identification of genetic variation in *Brassica napus* seeds for tocopherol content and composition using near-infrared spectroscopy technique. *Plant Breed.* 138 624–634. 10.1111/pbr.12708

[B41] Yan Y. Liang Y. Xuekun Z. Jingli G. Jijun W. (2021). Status and countermeasure of the high-quality development of rapeseed industy in China. *J. Agr. Sci. Tech Iran.* 23 1–7.

[B42] Yu L. Li G. Li M. Xu F. Beta T. Bao J. (2016). Genotypic variation in phenolic acids, vitamin E and fatty acids in whole grain rice. *Food Chem.* 197 776–782. 10.1016/j.foodchem.2015.11.027 26617016

[B43] Zaman Q. U. Chu W. Hao M. Shi Y. Sun M. Sang S. F. (2019). CRISPR/Cas9-mediated multiplex genome editing of JAGGED gene in *Brassica napus* L. *Biomolecules* 9:725. 10.3390/biom9110725 31726660 PMC6921047

[B44] Zaman Q. U. Wen C. Yuqin S. Mengyu H. Desheng M. Jacqueline B. (2021). Characterization of SHATTERPROOF homoeologs and CRISPR-Cas9-mediated genome editing enhances pod-shattering resistance in *Brassica napus* L. *CRISPR J.* 4 360–370. 10.1089/crispr.2020.0129 34152222

[B45] Zhai Y. Cai S. Hu L. Yang Y. Amoo O. Fan C. (2019). CRISPR/Cas9-mediated genome editing reveals differences in the contribution of INDEHISCENT homologues to pod shatter resistance in *Brassica napus* L. *Theor. Appl. Genet.* 132 2111–2123. 10.1007/s00122-019-03341-0 30980103

[B46] Zhai Y. Yu K. Cai S. Hu L. Amoo O. Xu L. (2020). Targeted mutagenesis of BnTT8 homologs controls yellow seed coat development for effective oil production in *Brassica napus* L. *Plant Biotechnol. J.* 18 1153–1168. 10.1111/pbi.13281 31637846 PMC7152602

[B47] Zhang H. Vasanthan T. Wettasinghe M. (2007). Enrichment of tocopherols and phytosterols in canola oil during seed germination. *J. Agric. Food Chem.* 55 355–359. 10.1021/jf060940o 17227065

