# Supplementary material for: Functional Differentiation of BnVTE4 Gene Homologous Copies in α-Tocopherol Biosynthesis Revealed by CRISPR/Cas9 Editing
Source: Front Plant Sci. 2022 Apr 11;13:850924. doi: 10.3389/fpls.2022.850924 (PMC9037293; doi:10.3389/fpls.2022.850924)
Supplement: Supplementary file 1 [file Data_Sheet_1.PDF]

Supplementary MaterialSupplementary Figures

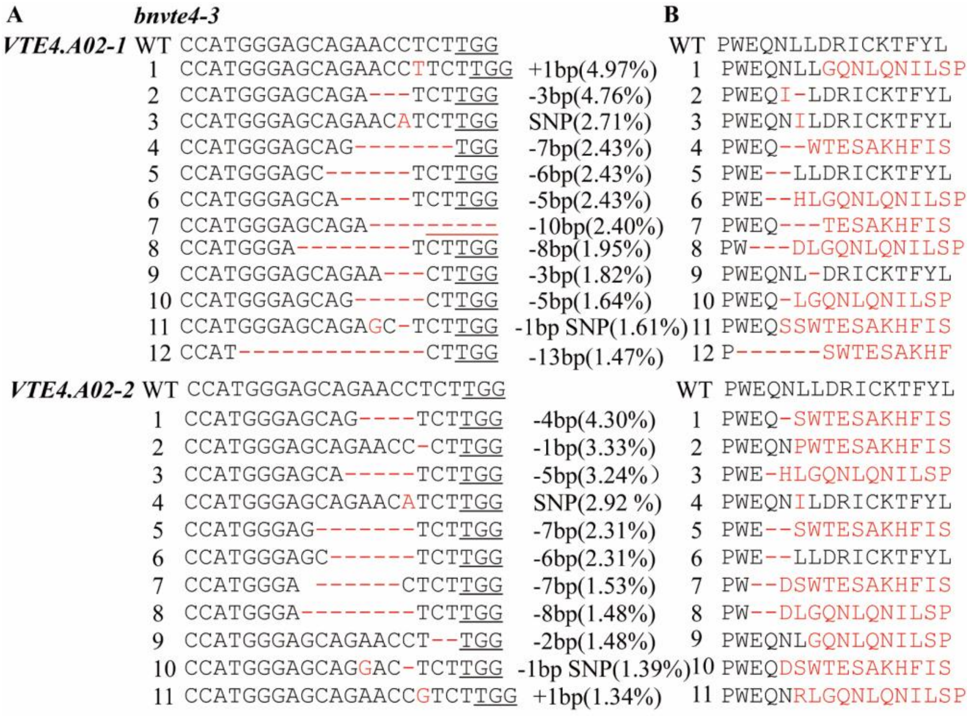

Supplementary Figure 1. Mutation types and amino acid changes in less than 5% of homologous copies of the *BnVTE4* gene in the T_1 generation mutant *bnvte4-3*. (A)： Mutation types and percentage of *VTE4.C02-1*, *VTE4.C02-2*, *VTE4.A02-1* and *VTE4.A02-2* in homologous copies of *BnVTE4* gene in *bnvte4-3* plants. (B): Amino acid alterations in different mutation types.

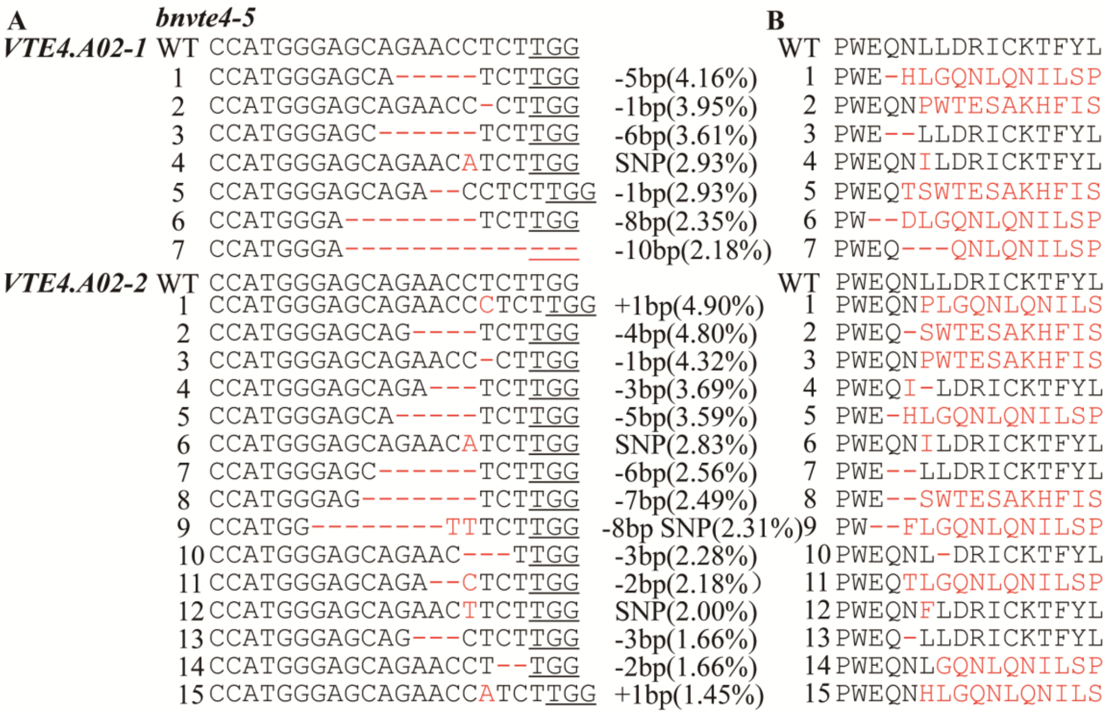

Supplementary Figure 2. Mutation types and amino acid changes in less than 5% of homologous copies of the *BnVTE4* gene in the T_1 generation mutant *bnvte4-5*. (A)： Mutation types and percentage of *VTE4.C02-1*, *VTE4.C02-2*, *VTE4.A02-1*and *VTE4.A02-2* in homologous copies of *BnVTE4* gene in *bnvte4-5* plants. (B): Amino acid alterations in different mutation types.

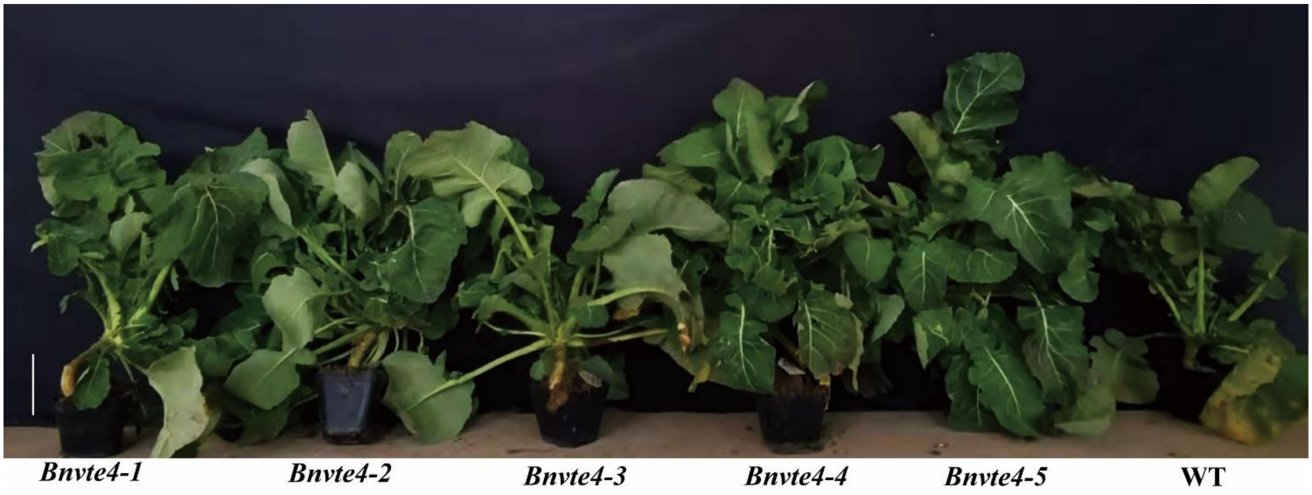

Supplementary Figure 3. Morphogenic of mutants and wild type(WT). Scale bar is 10cm.

Supplementary Table

Table 1. List of PCR primers

Primer name	Primer sequence(5'-3')	Product length
VTE4.C02-1 F	TAAACTGAAATAAAAATTTCGCAGTCG	1003bp
VTE4.C02-1 R	GATTTTAGCAATCGGAGCGTTAGAAAT	
VTE4.A02-1 F	GGTTGTTAATATTAATTTATATTATAAACACATGACTT	945bp
VTE4.A02-1 R	CTACTTACTGATTTTAGCATCGGAGCGT	
VTE4.A02-2 F	AATGAATAAGTTCCCCATTTCAAGATTAAG	1766bp
VTE4.A02-2 R	ATACTTACTGATTTTAGCATCGGAGCGT	
VTE4.A02-3 F	GTTGTTATTATTAATTTATATTATAAACACATGACTTTTT	940bp
VTE4.A02-3 R	AATTTTAGCAATTGGAGCGTGAGAAATAT	
hi*VTE4.A02-2* F	ggagtgagtacggtgtgcTGCCACAGAAATCTATCTCAAG	86bp
hi*VTE4.A02-2* R	gagttggatgctggatggGACATAATCAGTGGTGGAGCAC	
hi*VTE4.A02-3* F	ggagtgagtacggtgtgcCACAGAAATCTATCTCCAGGGG	80bp
hi*VTE4.A02-3* R	gagttggatgctggatggGACATAATCCGAGGTGGAGCAC	
